# Purification and properties of a novel quizalofop-*p*-ethyl-hydrolyzing esterase involved in quizalofop-*p*-ethyl degradation by *Pseudomonas* sp. J-2

**DOI:** 10.1186/s12934-017-0695-8

**Published:** 2017-05-10

**Authors:** Hui Zhang, Mengya Li, Jie Li, Guangli Wang, Yuan Liu

**Affiliations:** College of Life Sciences, Huaibei Normal University, Huaibei, 235000 China

**Keywords:** Quizalofop-*p*-ethyl, Biodegradation, *qpeH*, Metabolic pathway, *Pseudomonas* sp

## Abstract

Quizalofop-*p*-ethyl (QPE) is a post-emergence herbicide that effectively controls grass weeds and is often detected in the environment. However, the biochemical and molecular mechanisms of QPE degradation in the environment remains unclear. In this study, a highly effective QPE-degrading bacterial strain J-2 was isolated from acclimated activated sludge and identified as a *Pseudomonas* sp., containing the QPE breakdown metabolite quizalofop acid (QA) identified by Liquid Chromatography-Ion Trap-Mass Spectrometry (LC-IT-MS^n^) analysis. A novel QPE hydrolase esterase-encoding gene *qpeH* was cloned from strain J-2 and functionally expressed in *Escherichia coli* BL21 (DE3). The specific activity of recombinant QpeH was 198.9 ± 2.7 U mg^−1^ for QPE with *K*_*m*_ and *K*_*cat*_ values of 41.3 ± 3.6 μM and 127.3 ± 4.5 s^−1^. The optimal pH and temperature for the recombinant QpeH were 8.0 and 30 °C, respectively and the enzyme was activated by Ca^2+^, Cd^2+^, Li^+^, Fe^3+^ and Co^2+^ and inhibited by Ni^2+^, Fe^2+^, Ag^+^, DEPC, SDS, Tween 80, Triton X, β-mercaptoethanol, PMSF, and pCMB. In addition, the catalytic efficiency of QpeH toward different AOPP herbicides in descending order was as follows: fenoxaprop-P-ethyl > quizalofop-P-tefuryl > QPE > haloxyfop-P-methyl > cyhalofopbutyl > clodinafop-propargyl. On the basis of the phylogenetic analysis and multiple sequence alignment, the identified enzyme QpeH, was clustered with esterase family V, suggesting a new member of this family because of its low similarity of amino acid sequence with esterases reported previously.

## Background

Quizalofop-*p*-ethyl (QPE; ethyl(R)-2-[4-(6-chloroquinoxalin-2-yloxy) phenoxy] propionate) is a member of the aryloxyphenoxypropionate (AOPP) group of herbicides. Like other members of this family, QPE is a selective post-emergence herbicide that is registered for use in the control of annual and perennial grass weeds in crops of potatoes, soya beans, sugar beets, peanuts, oilseed rape, sunflowers, vegetables, cotton, flax, and other broad leafed plants [1, 2]. As a systemic herbicide that inhibits acetyl CoA carboxylase, QPE is absorbed from the leaf surface and translocated throughout the plant via the xylem and phloem, from the treated foliage to the root system, and accumulates in the meristematic tissue, thus inhibiting fatty acid biosynthesis [3].

The widespread use of QPE has led to detrimental effects on the environment. Several publications have reported that QPE and its metabolites have reproductive, genetic and liver toxicity [4, 5], as well as being a raw water contaminant [6]. Thus, the cleanup of QPE residues is an important part of environmental remediation [7]. The use of microbes and enzymes in the detoxification and decontamination of AOPP herbicides is considered a viable and environment-friendly approach. To date, only three QPE-degrading bacterial strains have been documented, including *Pseudomonas azotoformans* QDZ-1 [8], *Acinetobacter* sp. strain DL-2 [9], and *Rhodococcus ruber* JPL-2 [10]. Moreover, several genes encoding AOPP herbicide hydrolases that are involved in the degradation of AOPP herbicides have been identified [8–10]. To date, however, the metabolic pathway resulting in QPE degradation in microorganisms remains unclear and their QPE hydrolases have yet to be thoroughly investigated.

In the present study, we report the isolation and characterization of *Pseudomonas* sp. J-2, which can degrade QPE. For the first time, we also cloned and expressed a novel gene (*qpeH*) encoding a QPE hydrolase (QpeH) in *E. coli* BL21 (DE3). In addition, the characteristics of QpeH, including its substrate range and specificity were systematically investigated.

## Methods

### Chemicals

Quizalofop-*p*-ethyl (>97% purity) was kindly supplied by Fengle Agrochemical & Chemical Co., Ltd. (He fei, China). Quizalofop-P-tefuryl, fenoxaprop-P-ethyl, haloxyfop-P-methyl, cyhalofop-butyl, clodinafop-propargyl, diethyl pyrocarbonate (DEPC), phenylmethylsulfonyl fluoride (PMSF), 1,10-phenanthroline, and *N*-bromosuccinic acid (NBS) were purchased from Aladdin Industrial Inc. *p*-chloromercuribenzoic acid (pCMB) was purchased from the Shanghai Chemical Reagent Co., Ltd., China. All other chemicals and reagents were of analytical grade. Stock solutions of the above herbicides (40 mM) were prepared in dimethyl sulfoxide and sterilized by filtration with a pore size of 0.22 μm. Luria–Bertani (LB) medium and mineral salts medium (MSM) were used in this study [11].

### Isolation and identification of QPE-degrading bacteria

A soil sample (10 g) collected from the sewage outfall of the Fengle Agrochemical Co., Ltd. (He fei, China) was added to an Erlenmeyer flask (250 mL) containing 100 mL of MSM medium and QPE (20 mg L^−1^). After incubation for 7 days, the culture became turbid and 10 mL of the enrichment culture was transferred into another flask of fresh MSM medium containing 20 mg L^−1^ QPE. The rate of QPE removal was determined using high performance liquid chromatography (HPLC) as described below after the fourth transfer of the bacterial culture. A culture that degraded QPE was serially diluted and spread onto MSM agar plates containing 200 mg L^−1^ QPE. Colonies that grew on the plates were picked and purified by repeated streak plating. Isolates that possessed the highest QPE-degrading efficiency were selected and identified based on their morphological, physiological and biochemical properties combined with a 16S rRNA gene sequence analysis as described by Lin et al. [12].

### Chemical analysis and identification of metabolites

To determine the concentration of QPE, cells cultured in MSM were centrifuged (1370×*g*, 5 min) and the supernatants were extracted three times with an equal volume of dichloromethane. The aqueous layer was then acidified to pH 1.5–2.0 by the addition of 2 N hydrochloric acid and re-extracted three times with an equal volume of dichloromethane. The extracts were combined and dried over anhydrous sodium sulfate and evaporated under reduced pressure. The residues were dissolved in a minimal volume of methanol and the solution was filtered through a 0.22-μm Millipore membrane filter before analysis. All samples were analyzed by HPLC (Agilent 1260, USA) equipped with a C_18_ reverse phase column (150 mm × 4.6 mm × 5 μm) using a mobile phase consisting of methanol: water (80: 20 v/v) at a flow rate of 0.6 mL min^−1^ with an injection volume of 20 μL. The column was maintained at 25 °C, and the UV detector was set at 236 nm. The concentrations of other herbicides were determined according to the method of Nie et al. [8]. QPE metabolites were identified by a LC-IT-MS^n^ system (Liquid Chromatography-Ion Trap Mass Spectrometry, Thermo, USA) as described by Stolker et al. [13].

### Cloning of the QPE-hydrolyzing esterase gene and data analysis

General DNA manipulation was performed as described by Mamiatis et al. [14]. The QPE hydrolase gene from *Pseudomonas* sp. strain J-2 was cloned by first constructing a genomic DNA library using the shotgun method [9]. Bacterial genomic DNA was prepared using a high-salt extraction method [15] and subjected to partial digestion with the restriction enzyme *Sau*3AI. Fractions containing approximately 2- to 4-kb DNA fragments were isolated, purified, ligated into the *Bam*HI site of the cloning vector pUC118, and transformed into competent *E. coli* DH5α cells. Transformants were then plated onto LB agar plates supplemented with 100 mg L^−1^ of ampicillin and 200 mg L^−1^ QPE. The plates were incubated at 37 °C for approximately 10 h and stored at 16 °C for 48 h to allow transparent halos to be produced from QPE degrading colonies. Colonies exhibiting halos were picked and further tested by HPLC analysis for their QPE degradative ability. The selected positive clones were sequenced by Shanghai Sangon Biotech Co., Ltd. Nucleotide and deduced amino acid sequence analyses were performed using OMIGA 2.0 software (Oxford Molecular Ltd.). Blastn and Blastp tools (www.ncbi.nlm.nih.gov/Blast) were used for nucleotide sequencing and deduced amino acid sequence identity searches, respectively. Phylogenetic analysis of the protein sequences was performed using MEGA 6.0 software [16], and a bootstrap analysis including 1000 resamplings was used to evaluate the tree topology [17]. The presence of a signal peptide was predicted by the SignalP 4.1 server (http://www.cbs.dtu.dk/services/SignalP/) [18].

### Gene expression and purification of the recombinant QPE-hydrolyzing esterase

The *qpeH* gene was PCR-amplified from the genomic DNA of strain J-2 with PrimeSTAR HS DNA polymerase (TaKaRa) using the following primers: sense (5ʹ-TTT*GGATCC*ATGACCAAGATCTCTGCA-3ʹ), containing a *Bam*HI I site (underlined) corresponding to positions 1–18; and antisense (5ʹ-TTT*CTCGAG*GTCCTCGCGCTTCAGGAA-3ʹ), containing a *Xho*I site (underlined) after the stop codon and a 6× His tag before the stop codon.

The *qpeH* PCR product was digested with *Bam*HI and *Xho*I, and cloned into the expression vector pET-29a (+) to generate the recombinant plasmid pET-*qpeH*. QpeH was overexpressed in *E. coli* BL21 (DE3) using the His-Bind protein fusion and purification system following the method described by Wang et al. [15]. The protein concentration was determined using the Bradford method with bovine serum albumin as a standard [19].

### Determination of the molecular mass and p*I*

The molecular mass of denatured QpeH was determined by sodium dodecyl sulfate-polyacrylamide gel electrophoresis (SDS-PAGE) according to the method by Laemmli [20]. SDS-PAGE gels were stained with Coomassie blue G-250 (Amresco, USA). The molecular mass of the native protein was determined by gel filtration [21]. The p*I* of QpeH was predicted using PAGE with 6.25% Ampholine (pH 3.5–10.0) (GE Healthcare, Sweden) in a gel strip (0.5 cm × 1.0 cm) with an isoelectric focusing calibration kit (Pharmacia LKB Biotechnology), according to the supplier’s recommendations.

### Enzyme assay

All enzyme assays were performed in PBS (50 mM; pH 7.4), and no more than 10% of the substrate was hydrolyzed during each assay. For hydrolysis activity assays, 10 μL of QpeH (0.12 mg mL^−1^) was mixed with 0.08 mM substrate in 10 mL of PBS, and the reaction mixture was incubated at 37 °C for 1 min. One unit of QpeH activity was defined as the amount of enzyme required to hydrolyze 1 μmol of substrate per minute.

### Biochemical properties of the purified recombinant QpeH

The optimal reaction pH was determined by incubating the reaction mixtures at 37 °C in the following buffers: 20 mM citrate buffer, pH 3.0–6.0; 20 mM PBS, pH 6.0–8.0; 20 mM Tris–HCl buffer, pH 7.5–8.5; and 50 mM glycine-NaOH buffer, pH 8.5–11.0. The optimal reaction temperature was assessed using the optimal pH and incubating the reaction mixtures at different temperatures (20–65 °C) for 5 min.

To determine pH stability, the enzyme was pre-incubated at 4 °C for 24 h in different buffers and the residual activity was assayed using the assay conditions described above.

To determine thermostability, the enzyme was incubated at different temperatures and aliquots were withdrawn at specific time intervals, with the remaining activity determined as described above. Non-heated enzyme was used as a control (100%).

The effects of potential inhibitors or activators on the enzymatic activity of QpeH were analyzed by the addition of various metal salts and chemical agents to the reaction mixture, including Fe^2+^, Ba^2+^, Cu^2+^, Li^+^, Fe^3+^, Co^2+^, Ni^2+^, Zn^2+^, Ca^2+^, Mg^2+^, Cr^2+^, Ag^+^, and Mn^2+^ (1 mM); Tween-80, Triton X-100, SDS, EDTA, β-mercaptoethanol (10 mM), DEPC, PMSF, NBS, and pCMB (0.5 mM). The reaction mixtures were pre-incubated for 30 min at 37 °C with each inhibitor or activator and the enzymatic activity was assayed as described above. Enzyme activity without any additive was used as the control and was defined as 100%.

### Determination of kinetic constants

For kinetic studies, 0.8 μg of purified enzyme was assayed at varying substrate concentrations for each substrate (0.2–2.4 mM) at their optimal conditions. Kinetic parameters (*K*_*m*_ and *V*_max_) were calculated using a Lineweaver–Burk plot [22]. The specificity constant, *k*_cat_ */K*_*m*_, was calculated to determine the substrate specificity for each substrate. Each experiment was carried out in triplicate and a control experiment without QpeH was performed under the same conditions. Kinetic analyses were performed by curve fitting with the Sigma Plot Kinetic Module (version 1.3). The standard error was recorded to be <2%.

### Nucleotide sequence accession numbers

The nucleotide sequences of the 16S rRNA and the *qpeH* gene of *Pseudomonas* sp. strain J-2 have been deposited in the GenBank database (Accession Numbers KU522451 and KU522452, respectively).

## Results and discussion

### Isolation and characterization of the QPE-degrading strain

Quizalofop-*p*-ethyl has been widely used as an herbicide in the Anhui Province, China for many years. QPE residues have frequently been found in the soil; therefore, it is probable that several microbes may have adapted to this QPE-contaminated environment. After approximately 1 month of enrichment and isolation, four pure isolates that grew using QPE as the sole carbon and energy source were obtained from the acclimated activated sludge. The ability of these four strains to degrade QPE was confirmed in liquid MSM medium supplemented with QPE. One isolate, designated J-2, showed the highest QPE-degrading ability and was selected for subsequent experiments. Basic biochemical tests showed that strain J-2 consisted of Gram-negative rods that were positive for catalase-, oxidase, and nitrate reductase activity. Cells grew aerobically, were 1.8–2.0 μm long and 0.7–0.9 μm wide, and were motile with one polar flagellum. Optimal growth of strain J-2 was observed at pH 7.0, with a NaCl concentration of 0% (w/v), at 28 °C in LN medium (LB without NaCl) supplemented with 0–5% (w/v) NaCl. Phylogenetic analysis of the 16S rRNA gene sequence revealed that strain J-2 was closely affiliated with *Pseudomonas* sp. and was most closely related to *Pseudomonas zhaodongensis* with a similarity of 99.10% (data not shown).

*Pseudomonas* are ubiquitous and numerous in soil and can survive under extremely harsh conditions. Moreover, members of the genus *Pseudomonas* consists of a number of well-studied degraders of organic pollutants in the environment [23]. For example phenanthrene can be degraded by *Pseudomonas* sp. JM2 [23] and *Pseudomonas stutzeri* strain ZP2 [24], nicotine by *Pseudomonas* sp. strain HF-1 [25], pyrene by *Pseudomonas* sp. strain Jpyr-1 [26], and sulfadoxine by *Pseudomonas* sp. DX7 [27]. In this study, a pure bacterial strain identified to the genus *Pseudomonas* was isolated and efficiently degraded QPE. These features make them ideal candidates for the bioremediation of contaminated environments.

### Cloning and sequence analysis of the QPE-hydrolase gene

To clone the QPE-hydrolase gene, a genomic library of strain J-2 was constructed as previously described [15] and one positive clone that produced a transparent halo around the colony was identified from approximately 20,000 transformants. The ability of this strain to degrade QPE was confirmed by HPLC and a plasmid from this strain was extracted and subjected to DNA sequencing. The results showed that the inserted fragment in the transformant was 2086 bp long and contained two complete ORFs. The two identified ORFs were then subcloned into the vector pMD18-T and transformed into *E. coli* DH5α. One of the ORFs was identified as the structural gene encoding QPE hydrolase and was designated as *qpeH*.

The cloned *qpeH* gene was 930 bp in length with a GC content of 70.1% and encoded a protein of 309 amino acids with a calculated molecular mass of 32,831 Da. A putative signal peptide was identified at the N terminus by using the SignalP 4.1 server, with the most likely cleavage site situated between amino acids Ala33 and Gly34, resulting in a putative 276-residue mature protein. The corresponding protein was used as a query sequence in a homology search against the Protein Data Bank (NCBI database). This search revealed that the most closely related proteins were several hypothetical proteins (hydrolases or esterases); for example, α/β hydrolase from *Brevundimonas diminuta* (89%), esterase sys410 from uncultured bacterium (76%), and lactofen hydrolase from *Brevundimonas* sp. LY-2 (72%). Recently, cloning of the novel gene *chbH,* encoding a cyhalofop-butyl hydrolase, from *P. azotoformans* strain QDZ-1 was reported [8]. ChbH hydrolyzed all AOPP herbicides tested, including QPE, with a specific activity of 4.12 ± 0.25 μmol min^−1^ mg^−1^ [8]. A novel carboxylesterase encoding gene (*feh*) was cloned from *R. ruber* strain JPL-2 and FeH had a broader substrate spectrum and higher catalytic efficiency toward AOPP herbicides [10]. In the same year, another novel FE hydrolase/esterase, AfeH, which also hydrolyzed various AOPP herbicides and belonged to family VII of lipolytic enzymes, was characterized [9]. In the present study, QpeH from strain J-2 had 7.9 and 9.3% similarity to that of FeH and ChbH, respectively, but only showed 5.8% similarity to AfeH.

**Fig. 1:**
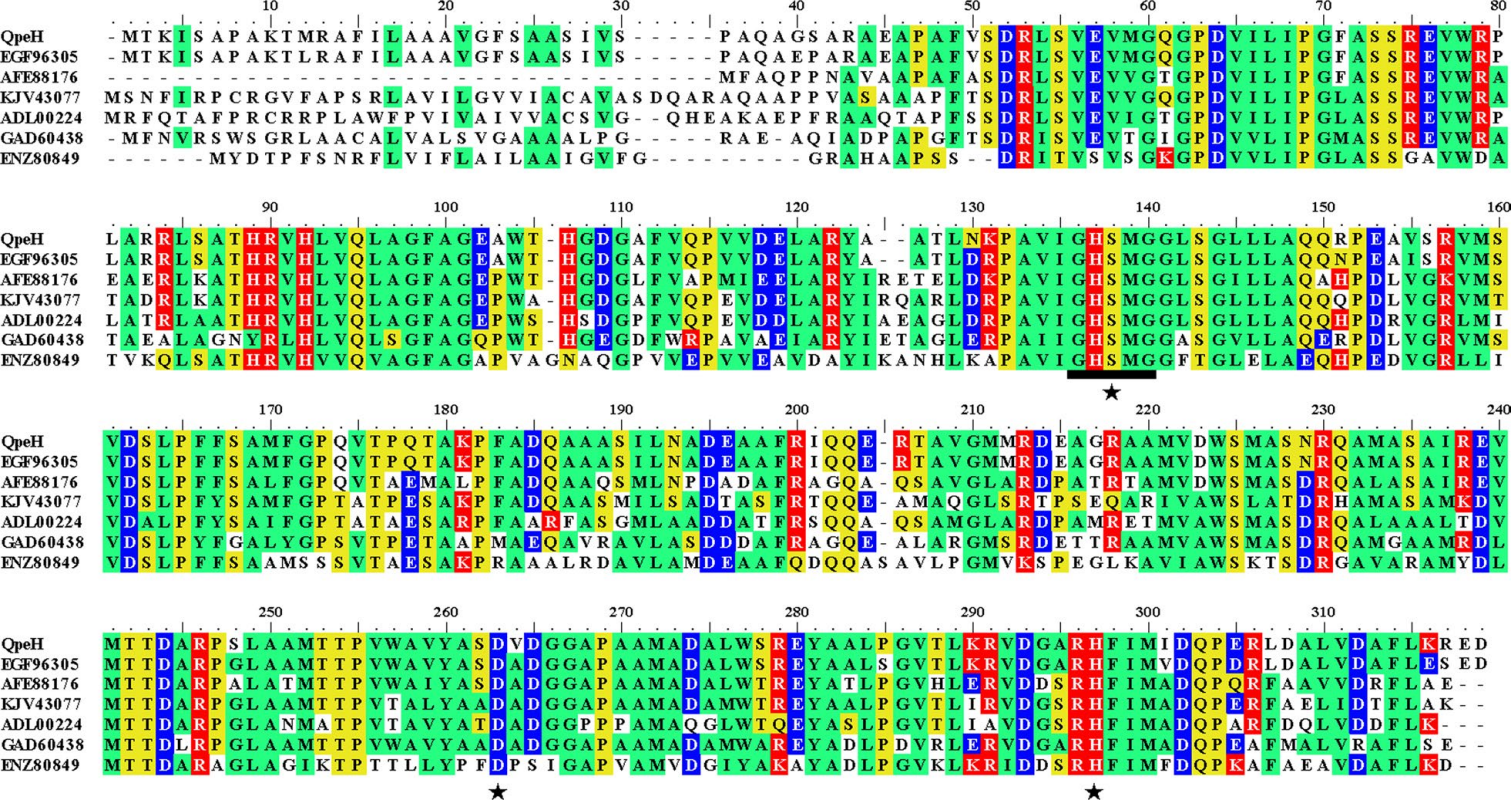
Sequence alignment of QpeH with the most closely related proteins available in the Protein Data Bank (PDB): EGF96305, alpha/beta hydrolase fold family protein of *Brevundimonas diminuta* ATCC 11568; AFE88176, esterase sys410 of uncultured bacterium; KJV43077, alpha/beta hydrolase of *Brevundimonas* sp. KM4; ADL00224, alpha/beta hydrolase fold protein of *Brevundimonas subvibrioides* ATCC 15264; GAD60438, alpha/beta hydrolase fold family of *Brevundimonas abyssalis* TAR-001; and ENZ80849, putative hydrolase of *Caulobacter crescentus* OR37. The conserved hydrolase motif (G-X-S-X-G) is underlined, and the amino acids that form the catalytic triad (Ser129, Asp255, and His289) are indicated by *asterisks*. Similar and identical amino acid residues are shown in *color* (non polar, neutral, positive, and negative amino acid residues are shown in *green*, *yellow*, *red* and *blue*, respectively). *Numbers* above amino acid residues indicate amino acid positions

**Fig. 2:**
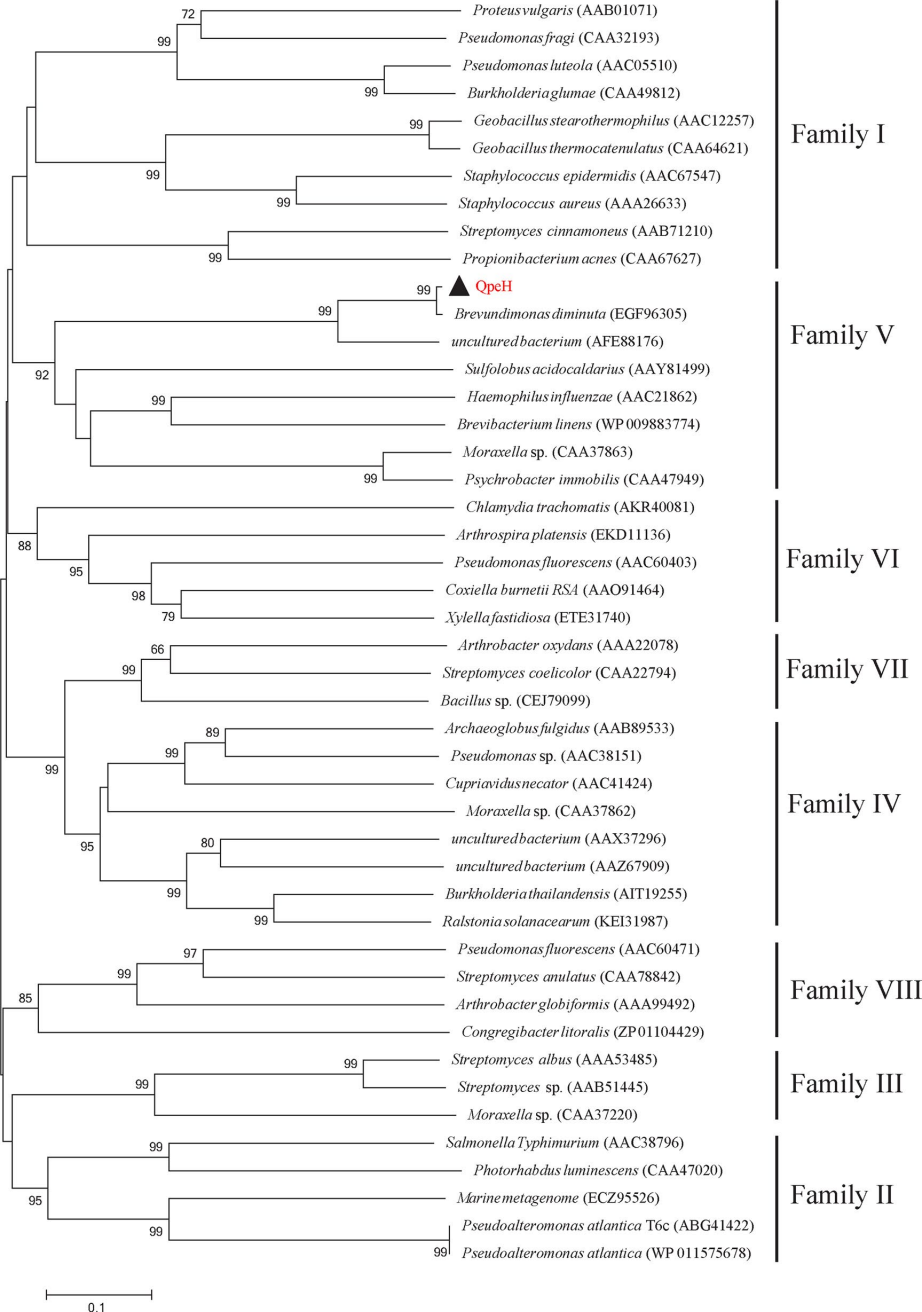
Phylogenetic analysis of QpeH demonstrating its relation to other esterases from subfamilies I–VIII. The phylogenetic tree was constructed using the neighbor-joining method with 1000 bootstrap replicates (values >60% are shown at the nodes). The related protein sequences retrieved from GenBank were aligned using ClustalW Bar, 0.1

QpeH also contained the conserved esterase family sequence motif (G-X-S-X-G) (Fig. 1) [28]. Moreover, a catalytic triad that is also highly conserved in this enzyme family [29], Ser-Asp-His (Ser129, Asp255, and His289), was also identified. Bacterial lipase/esterase proteins have been classified into eight different families based on their amino acid sequences and biochemical properties [30]. A phylogenetic tree was constructed to verify the evolutionary relationship between QpeH and its closest relatives, as well as to characterize members of the esterase family [31]. Based on these data, QpeH was shown to be a novel QPE hydrolase that belongs to family V, and which is completely different from FeH from *R. ruber* strain JPL-2, ChbH from *P. azotoformans* QDZ-1 and AfeH from *Acinetobacter* sp. strain DL-2, based on amino acid sequence comparisons and phylogenetic analyses (Fig. 2).

### Expression and purification of recombinant QpeH

**Fig. 3:**
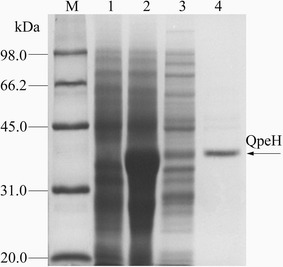
SDS-PAGE analysis of purified QpeH stained with Coomassie brilliant blue G250. *Lane M*, protein molecular mass marker; *lane 1*, *E. coli* BL21 (DE3) pET-29a (+) whole cell lysate; *lane 2*, *E. coli* BL21 (DE3) pET-29a-*qpeH* whole cell lysate; *lane 3*, soluble proteins of *E. coli* BL21 (DE3) pET-29a-*qpeH*; *lane 4*, purified QpeH

**Fig. 4:**
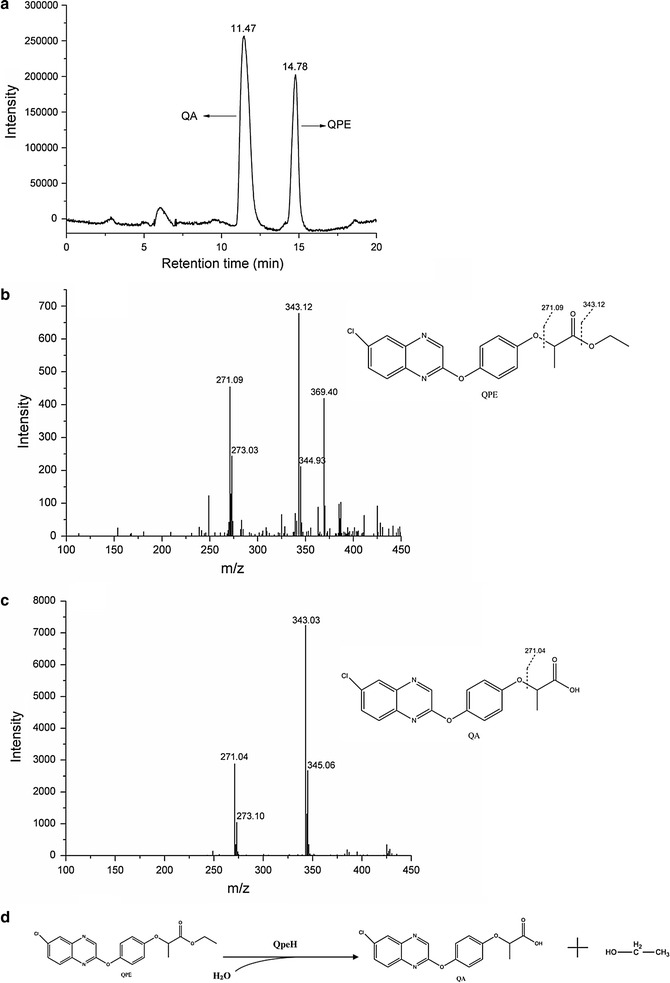
LC–MS analysis of QPE intermediates resulting from QpeH hydrolysis of the substrate and a proposed metabolic pathway. **a** LC profiles for QPE and its intermediate identified as QA. **b**–**c** Mass spectrum of characteristic ions for QPE (RT = 14.78 min) and the metabolite QA (RT = 11.47 min). **d** Proposed pathway of QPE hydrolysis by QpeH. QPE is converted to QA and ethanol by hydrolysis

Recombinant QpeH was produced in *E. coli* BL21 (DE3) and purified from the crude extract using Ni-nitrilotriacetic acid affinity chromatography. The purified enzyme gave a single band on SDS-PAGE (Fig. 3). The molecular mass of recombinant QpeH was approximately 39 kDa, which was consistent with the molecular mass deduced from the amino acid sequence (38,500 Da). The estimated molecular mass of the native enzyme was also approximately 39 kDa as determined by gel filtration, which suggested that the enzyme was a monomer. Furthermore, the p*I* value of QpeH was 6.7 by isoelectric focusing. QpeH was similar in size to other reported hydrolases, such as AfeH (34 kDa) from *Acinetobacter* sp. strain DL-2 [9], ChbH (36 kDa) from *P. azotoformans* strain QDZ-1 [8] and PytH (31 kDa) from *Sphingobium* sp. strain JZ-1 [32]. In addition, the metabolites were identified by LC-IT-MS^n^. These results indicated that QpeH catalyzed the hydrolysis of QPE to quizalofop acid (QA) and ethanol (Fig. 4).

### Characteristics of QpeH

**Fig. 5:**
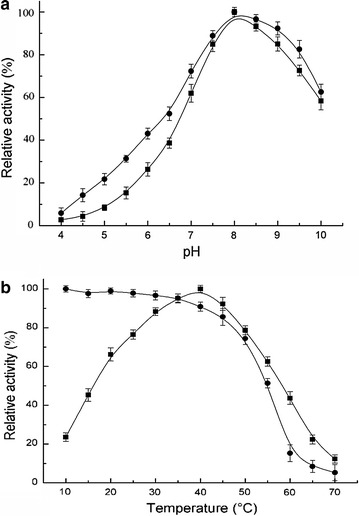
Effects of pH (**a**) and temperature (**b**) on QpeH activity (*closed squares*) and stability (*closed circles*). QPE was used as the substrate in all reactions. The values are shown as a percentage of the maximum activity (100%)

Environmental factors such as pH and temperature can affect enzyme activity and stability [33]. The recombinant QpeH exhibited high levels of activity at pH 7.5–8.5, with an optimum pH of 8.0 (Fig. 5a). Little activity was detected at pH values below 4.0 or above 13.0. The enzyme retained more than 85% of the original activity after pre-incubation in a buffer at a pH range below 4.0 or above 13.0 for 1 h (Fig. 5a).

The enzyme was active at 20–65 °C, with an optimum temperature of 30 °C (Fig. 5b). The thermal stability of the purified enzyme was assayed by incubating QpeH for 10 h between 20 and 45 °C in the absence of QPE (Fig. 5b). QpeH was stable and retained >50% of its residual activity after 10 h at temperatures <30 °C, but was unstable at temperatures >50 °C, suggesting QpeH is a mesophilic enzyme. In this study, QpeH exerted high activity and stability over a broad pH and temperature range.

The substrate specificity of QpeH was assessed with various aryloxyphenoxy propanoate AOPP herbicides as substrates (Table 1). QpeH hydrolyzed all AOPP herbicides tested, with different hydrolysis rates in descending order as follows: fenoxaprop-P-ethyl > quizalofop-P-tefuryl > QPE > haloxyfop-P-methyl > cyhalofop-butyl > clodinafop-propargyl (Table 1). This result indicated that the enzyme has potential applications in the in situ bioremediation of AOPP herbicide residues. The specific activity of QpeH for different herbicides was 198.9 ± 2.7 U mg^−1^ for QPE and 257.2 ± 3.7 U mg^−1^ for fenoxaprop-P-ethyl. The most suitable substrate was fenoxaprop-P-ethyl with *K*_*m*_ and *K*_*cat*_ values of 26.9 ± 4.3 µM and 161.6 ± 3.4 s^−1^, respectively. The specific activities of other enzymes for fenoxaprop-P-ethyl were 1080 U mg^−1^ for FeH and 3.95 ± 0.21 mg^−1^ for the cyhalofop-butyl-hydrolyzing esterase, ChbH [8, 10].

**Table 1:** Kinetic parameters for the hydrolysis of various AOPP herbicides

	*K*_m_ (µM)	*K*_cat_ (s^−1^)	*K*_cat_/*K*_m_ (s^−1 ^µM^−1^)	Enzyme activity (U/mg)
QPE	41.3 ± 3.6	127.3 ± 4.5	3.08 ± 0.03	198.9 ± 2.7
Quizalofop-P-tefuryl	29.5 ± 2.7	146.4 ± 3.3	4.96 ± 0.09	238.1 ± 4.1
Fenoxaprop-P-ethyl	26.9 ± 4.3	161.6 ± 3.4	6.01 ± 0.05	257.2 ± 3.7
Haloxyfop-P-methyl	113.9 ± 3.8	67.4 ± 2.6	0.59 ± 0.04	64.7 ± 2.4
Cyhalofop-butyl	158.2 ± 3.5	41.1 ± 4.1	0.26 ± 0.03	48.6 ± 3.1
Clodinafop-propargyl	174.0 ± 4.2	37.1 ± 2.9	0.21 ± 0.02	42.3 ± 2.8

Metal ions often play a key role in the activity of enzymes. As shown in Table 2, 1 mM Ca^2+^ and Cd^2+^ strongly stimulated QpeH enzyme activity, while Li^+^, Fe^3+^ and Co^2+^ also slightly increased enzymatic activity. However, Ba^2+^ and Ag^+^ reduced QpeH activity slightly, while Ni^2+^, Fe^2+^, and Ag^+^ were strongly inhibitory for QpeH. Cu^2+^, Mn^2+^, and Zn^2+^ ions had little effect on the enzyme activity at a concentration of 1 mM.

**Table 2:** Effect of metal ions on QpeH enzyme activity

Substances	Relative activity (%)	Substances	Relative activity (%)
None	100	1 mM Ba^2+^	86 ± 1.7
1 mM Ca^2+^	167 ± 2.1	1 mM Mn^2+^	91 ± 1.6
1 mM Ni^2+^	63 ± 1.5	1 mM Li^+^	122 ± 2.9
1 mM Cu^2+^	97 ± 1.3	1 mM Zn^2+^	97 ± 1.5
1 mM Fe^2+^	72 ± 2.3	1 mM Cd^2+^	152 ± 1.6
1 mM Fe^3+^	116 ± 2.2	1 mM Co^2+^	113 ± 2.2
1 mM Mg^2+^	80 ± 1.8	1 mM Ag^+^	36 ± 1.3

The effect of various chemical agents on enzyme activity was also investigated. QpeH had no apparent requirement for metal ions because the chelating agents EDTA and 1, 10-phenanthroline had little effect on enzyme activity [32]. Treatment of QpeH with 10 mM DEPC resulted in a complete loss of catalytic activity, which indicated the involvement of His residues in the active sites of the enzyme [34]. The surfactants SDS, Tween 80, Triton X 100 (10 mM), and β-mercaptoethanol (10 mM) all strongly inhibited QpeH activity, as did the Ser protease inhibitor PMSF and the thiol reagent pCMB (10 mM). Chemical modification of QpeH with 10 mM NBS resulted in a 70% inhibition of catalytic activity (Table 3).

**Table 3:** Effect of chemical agents on QpeH enzyme activity

Substances	Relative activity (%)	Substances	Relative activity (%)
None	100	10mM1, 10-phenanthroline	96 ± 1.4
10 mM EDTA	93 ± 0.3	10 mM NBS	29 ± 2.1
10 mM β-mercaptoethanol	43 ± 1.3	10 mM PCMB	35 ± 2.2
10 mM Tween 80	13 ± 0.9	10 mM PMSF	23 ± 1.7
10 mM SDS	7.7 ± 1.7	10 mM DEPC	0.5 ± 0.2
10 mM Triton X 100	3.1 ± 1.1		
